# Adalimumab-induced hypersensitivity pneumonitis: a rare reaction to a common dermatologic drug

**DOI:** 10.36416/1806-3756/e20250478

**Published:** 2026-05-22

**Authors:** Miguel Ribeiro, Sofia Lopes, Catarina Cerqueira, Carlos Manuel Nogueira, Luís Reis, Celeste Brito, Márcio Rodrigues, Rui Rolo, Eva Dias Padrão

**Affiliations:** 1 Departamento de Dermatologia, Hospital de Braga, Braga, Portugal.; 2 Departamento de Radiologia, Hospital de Braga, Braga, Portugal.; 3 Departamento de Pneumologia, Hospital de Braga, Braga, Portugal.

## TO THE EDITOR:

TNF-α inhibitors are widely used for the treatment of chronic inflammatory diseases, including multiple dermatologic disorders. Adalimumab is one of the most frequently used biologic agents in moderate-to-severe plaque psoriasis, with proven effectiveness in cutaneous and articular manifestations.^1^ ^,^ ^2^ Drug-induced interstitial lung diseases (ILDs), including nonspecific interstitial pneumonia, organizing pneumonia, and pulmonary fibrosis, are rare; however, they are associated with anti-TNF therapy, most frequently in patients with rheumatoid arthritis or inflammatory bowel disease.^3^ ^-^ ^6^ Although TNF-α has yet to be completely understood, it appears to have profibrotic and antifibrotic activity in the lung, a paradox that might explain conflicting reports of anti-TNF agents having therapeutic and causative effects on ILD.^5^

Drug-induced hypersensitivity pneumonitis (HP) is a rare but clinically significant form of ILD in which systemic drugs act as antigenic triggers, eliciting an immune-mediated interstitial inflammatory response. However, to date, there have been no published cases of drug-induced HP during adalimumab therapy. Here, we describe what appears to be the first documented case of adalimumab-induced nonfibrotic HP in a patient with psoriasis, highlighting the need for clinical vigilance regarding pulmonary adverse events, even in patients without prior lung disease and long-term biologic therapy. 

We report the case of a 49-year-old male former smoker who was previously healthy and who presented with nonspecific respiratory symptoms, including dry cough and mild exertional dyspnea. He was a construction worker living in a rural environment with long-standing seasonal exposure to mold. He was being followed for plaque psoriasis and had been receiving 40 mg of adalimumab every two weeks for over two years, with stable disease control. No additional medications or systemic symptoms were reported. 

**Figure 1:**
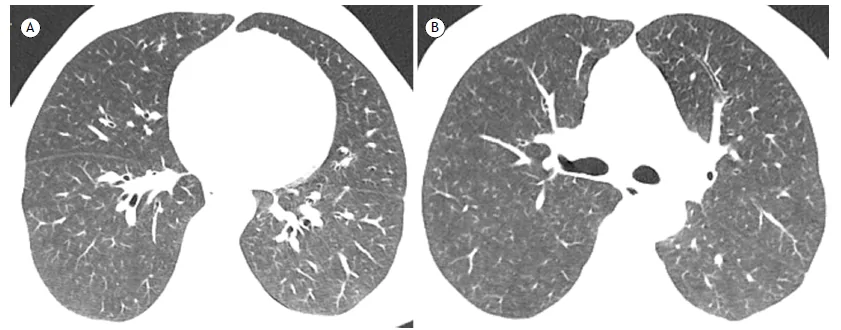
Chest HRCT scans showing bilateral diffuse centrilobular ground-glass micronodules (in A and B).

During screening for latent tuberculosis infection, a routine chest X-ray revealed a subtle hypotransparency in the upper third of the right hemithorax. Chest HRCT subsequently showed bilateral diffuse centrilobular ground-glass micronodules (Figure 1). BAL showed a total cellularity of 389 cells/µL, with marked lymphocytosis (72%). Laboratory workup, which included a complete blood count, as well as renal and liver panels, together with inflammatory markers and autoimmune screening, was unremarkable, and cultures were negative. The combination of imaging and BAL findings was consistent with nonfibrotic HP. However, the patient had not changed his environmental exposure. His long-standing, intermittent exposure to mold was due to a seasonal indoor activity performed every year. In this context, drug-induced pulmonary toxicity was strongly suspected. 

**Figure 2:**
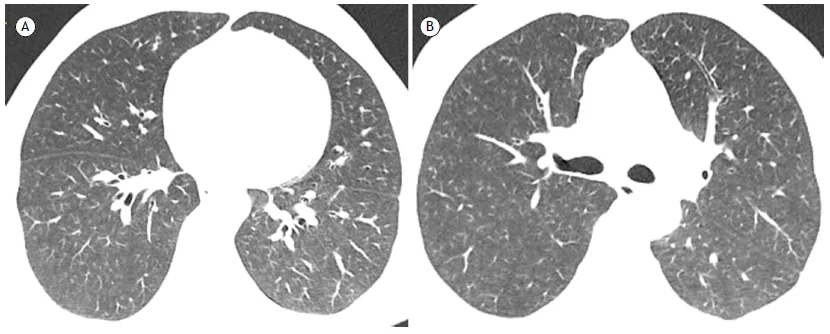
Follow-up chest HRCT scans showing resolution of the ground-glass micronodules after adalimumab discontinuation (in A and B).

Adalimumab was discontinued and switched to ixekizumab, and a complete resolution of respiratory symptoms was documented soon after withdrawal, without the need for corticosteroids. There was no recurrence of symptoms during later assessment. A follow-up chest HRCT scan performed several months later showed radiographic resolution of the findings (Figure 2), although the environmental exposure remained unchanged. 

Adalimumab is a fully human anti-TNF-α monoclonal antibody that has demonstrated efficacy and safety in psoriatic arthritis and moderate-to-severe plaque psoriasis. Clinical efficacy in psoriasis has been demonstrated by at least 75% improvement in the Psoriasis Area and Severity Index score at 16 weeks^7^ and significant improvements in joint, skin, and function endpoints in psoriatic arthritis, as well as rapid and sustained American College of Rheumatology 20% improvement responses.^2^ Established safety has been consistently reaffirmed across multiple studies and patient populations.^1^ Despite the long-standing safety and efficacy of anti-TNF agents in autoimmune diseases, increasing consideration has been given to their potential pulmonary adverse reactions, most prominently ILD.^4^ ^,^ ^8^ While some studies report no significant association,^9^ others suggest an apparent increased risk, especially among patients with previous lung disease or rheumatoid arthritis.^4^ Additional evidence comes from case reports describing acute ILD temporally related to adalimumab, including the first case reported in Brazil in a patient with rheumatoid arthritis and no prior lung disease.^10^ Reports of ILD in psoriasis patients are rare, particularly in the absence of joint involvement or comorbidities. One prior case described delayed onset ILD during long-term adalimumab therapy, with symptom and imaging resolution after drug withdrawal.^11^

In the pathogenesis of drug-induced HP there is an immunologically mediated T cell process involving alveolar inflammation, granuloma formation, and potential progression to fibrosis.^6^ ^,^ ^12^ Methotrexate-, minocycline-, and rituximab-associated cases have all presented with alveolar lymphocytosis and clinical recovery after discontinuation of the offending agent, supporting a reversible immune reaction.^13^ ^-^ ^15^ CD8+ T cell pathogenicity and cytotoxicity against drug-containing alveolar macrophages have indeed been proposed.^13^ Similarly, adalimumab may act as an antigenic trigger in predisposed patients, eliciting a hypersensitivity reaction and leading to drug-induced HP. In the case reported here, resolution of respiratory symptoms and radiographic findings after withdrawal of adalimumab, despite unchanged environmental exposure, strongly supports a drug-induced mechanism. A Naranjo score of 6, indicating a probable adverse drug reaction, further reinforces this association. 

The case reported here highlights a rare but likely underrecognized pulmonary adverse reaction to adalimumab in a dermatology setting. Drug-induced HP should be included in the differential diagnosis of new respiratory symptoms in patients on long-term biologic therapy. Prompt recognition and removal of the offending agent may lead to complete recovery without the need for additional treatment. 
